# Nutritional parameters predicting pressure ulcers and short-term mortality in patients with minimal conscious state as a result of traumatic and non-traumatic acquired brain injury

**DOI:** 10.1186/s12967-015-0660-4

**Published:** 2015-09-17

**Authors:** Tiziana Montalcini, Marta Moraca, Yvelise Ferro, Stefano Romeo, Sebastiano Serra, Maria Girolama Raso, Francesco Rossi, Walter G. Sannita, Giuliano Dolce, Arturo Pujia

**Affiliations:** Clinical Nutrition Unit, Department of Medical and Surgical Science, University Magna Grecia, Viale S. Venuta, 88100 Catanzaro, Italy; Department of Molecular and Clinical Medicine, Sahlgrenska Center for Cardiovascolar and Metabolic Research, University of Gothenburg, Gothenburg, Sweden; S.Anna Neurological Institute, Crotone, Italy

**Keywords:** Malnutrition, Minimal conscious state, Pressure ulcer, Mid-arm circumference, Albumin

## Abstract

**Background:**

The association between malnutrition and worse outcomes as pressure ulcers and mortality is well established in a variety of setting. Currently none investigation was conducted in patients with long-term consequences of the acquired brain injury in which recovery from brain injury could be influenced by secondary complications. The aim of this study was to investigate the association between various nutritional status parameters (in particular albumin) and pressure ulcers formation and short-term mortality in minimal conscious state patients.

**Methods:**

In this prospective, observational study of 5-months duration, a 30 patients sample admitted to a Neurological Institute was considered. All patients underwent a complete medical examination. Anthropometric parameters like mid-arm circumference and mid-arm muscle circumference and nutritional parameters as serum albumin and blood hemoglobin concentration were assessed.

**Results:**

At univariate and logistic regression analysis, mid-arm circumference (p = 0.04; beta = −0.89), mid-arm muscle circumference (p = 0.050; beta = −1.29), hemoglobin (p = 0.04, beta −1.1) and albumin (p = 0.04, beta −7.91) were inversely associated with pressure ulcers. The area under the ROC curve for albumin to predict sores was 0.76 (p = 0.02) and mortality was 0.83 (p = 0.03). Patient with lower albumin had significantly higher short-term mortality than those with higher serum albumin (p = 0.03; χ^2^ test = 6.47).

**Conclusion:**

Albumin, haemoglobin and mid-arm circumference are inversely associated with pressure ulcers. Albumin is a prognostic index in MCS patients. Since albumin and haemoglobin could be affected by a variety of factors, this association suggests to optimize nutrition and investigate on other mechanism leading to mortality and pressure ulcers.

## Background

Several studies have suggested that hospitalized patients with a poor nutritional status experience worse outcomes such as prolonged length of stay, pressure ulcers and mortality in comparison to those well-nourished [1–4]. Unfortunately, malnutrition is a common problem in the hospital setting, affecting about 50 % of patients [5].

Serum albumin and blood haemoglobin concentration are commonly used as indexes of nutritional status, although both have several weakness [6, 7].

Hypoalbuminemia has been shown to increase mortality in different clinical settings [8–10], including ischemic stroke [11, 12] and traumatic brain injury [13–15] while a decreased hemoglobin level was significantly associated with pressure ulcer formation in individual with traumatic brain injury [16, 17].

Although this issue has been widely studied especially in the first weeks or months after a brain injury [13, 14, 16], there is currently limited information concerning patients with chronic disorders of consciousness (CDC) that are the long-term consequences of traumatic and non-traumatic acquired brain injury. In particular, studies investigating the role of various biochemical and anthropometric indexes of nutritional status in predicting pressure ulcer and mortality are lacking. This is an important issue since the development of secondary complications may affects recovery from brain injury [18]. Furthermore, among CDC patients, those with minimal conscious state (MCS) are characterised by minimal fluctuating awareness with possible perception of suffering [19]. Consequently, in these patients pressure ulcer prevention is a priority but investigations on the factors contributing to pressure ulcer development in these patients are lacking.

The aim of this investigation was to identify the biochemical and anthropometric indexes of nutritional status associated to pressure ulcers and to verify whether albumin predicts short-term mortality in MCS patients.

## Methods

### Design

In this prospective, observational study of 5-months duration starting in November 2013, we enrolled patients diagnosed with MCS who were admitted to a Neurological Institute.

### Setting and sample

A convenience sample of 30 patients admitted to a Neurological Institute in Crotone, Italy, fulfilled all criteria and participated in the study (age range of 45–76 years; 14 female). They were also all those who, at the time of the study, were hospitalized in the part of the Institute dedicated to MCS patients (acute patients were hospitalized in a different building Institute).

Patients were eligible for inclusion if they met the standard clinical diagnostic criteria for MCS [20]. These patients show at least some subtle and intermittent signs of awareness (voluntary motor activity like fixating and tracking objects with their eyes) and may follow simple commands, but are usually unable to uphold a meaningful communication and interaction with their environment [20].

Exclusion criteria were as follows: premorbid history of developmental, psychiatric or neurological illness, spinal cord injury, co-existing systemic disease with a limited life expectancy.

The investigation was carried out in agreement with the Helsinki Declaration and all laws and relative to patients’ rights and was approved by the local ethic committee. Written informed consent was obtained from each patient’s legal surrogate (for all participants).

### Definitions

The European Pressure Ulcer Advisory Panel (EPUAP) description was used to define the pressure ulcers as “an area of localized damage to the skin and underlying tissue caused by pressure, shear, friction and/or a combination of these things” [21]. We used the following classification also developed by EPUAP [9]:

- Grade 1: Nonblanchable erythema of intact skin.
- Grade 2: Partial thickness skin loss involving epidermis and/or dermis: the pressure ulcer is superficial and presents clinically as an abrasion, blister or shallow crater.
- Grade 3: Full-thickness skin loss involving damage or necrosis of subcutaneous tissue that may extend down to, but not through, underlying fascia: the pressure ulcer presents clinically as a deep crater with or without undermining of adjacent tissue.
- Grade 4: Extensive destruction tissue necrosis, or damage to muscle, bone or supporting structures with or without full-thickness skin loss.

### Data collection

The nutritional state was assessed by a trained dietitian while pressure ulcer development by internists according to the study protocol and neurological signs, such as spastic hypertonia or seizures signs, by neurologists. Data collection was performed by dietitian. No particular nutritional scheme was pursued in the Institute apart the administration of 1.3–1.5 g/kg of proteins at each patients.

#### Caloric intake and anthropometric measurements

The participant’s caloric intake was deduced from the medical record. Body weight was measured with a calibrated lift scale in the morning, with the subjects lightly dressed, subtracting the weight of clothes. Height was estimated from knee height by a validated equation for mobility-impaired individuals [22]. BMI was calculated with the following equation: weight (kg)/height (m^2^).

Skin fold thickness was measured three times at the triceps (TSF) with the GIMA Skinfold Caliper (Gessate, Milan, Italy) [23, 24] and the mean was calculated.

Mid-arm circumference (MAC), a measures of muscle mass, was assessed at the level of the mid-point between the acromiale (lateral edge of the acromion process, e.g. bony tip of shoulder) and the radiale (proximal and lateral border of the radius bone, approximately the elbow joint), on the mid-line of the posterior surface of the arm [25]. Mid-arm muscle circumference (MAMC) was calculated as follow [25]:

$${\text{MAMC }}\left( {\text{cm}} \right) = {\text{MAC }}\left( {\text{cm}} \right) - \left( {\pi \times {\text{TSF}}} \right)$$

All assessments were performed by a dietitian.

#### Instrumental measurements

Body composition was estimated by using a bioelectrical impedance analysis (BIA) (BIA-101, Akernsrl, Florence, Italy) to calculate the Total Body Water (TBW), Fat Mass (FM), Muscle Mass (MM), total Fat-Free Mass (FFM), extra-cellular water (ECW), intra-cellular water (ECW) and phase angle (PA) [26]. Since participants were unable to separate the limbs effectively, an insulating barrier (dry clothes) was provided [27].

Energy needs were calculated with the Harris-Benedict equation [28].

The estimation of total energy expenditure (TEE) was performed by SenseWear Armband (Pro3; BodyMedia, Inc., Pittsburgh, PA, USA), a wireless multisensory activity monitor normally worn on the upper right arm over the triceps muscle, halfway between the acromion and olecranon [29]. These last two measurements were performed to compare the calories needs with the real caloric intake chosen by physicians.

The body composition assessment was performed by a dietitian and TEE estimation by a biotechnologist.

### Biochemical evaluation

Venous blood was collected in the morning into vacutainer tubes (Becton & Dickinson) and centrifuged within 2 h. Serum glucose, creatinine, calcium, iron (v.n. female 20–140 mcg/dl and male 60–160 mcg/dl), transferrin (v.n. 250–400 mg/dl), albumin (n.v. 3.70–5.30 g/dl) were measured using the commercially available ELISA assay kits. Red blood count (RBC) and white blood count (WBC) were measured with flow cytometry, hemoglobin (Hgb-n.v. male 12.9–17.7 g/dL, female 10.5–15.3 g/dL) with Spectrophotometric assay. Quality control was assessed daily for all determinations.

### Statistical analysis

Data are reported as mean ± standard deviation (SD). ANOVA test was used to compare the means between groups categorized by albumin level (group I: concentration lower than 3 g/dl; group II: 3 g/dl <albumin <3.5 g/dl; group III: equal or more than 3.5 g/dl); A χ^2^ test was performed to analyze the difference in the prevalence between groups. Pearson correlation was used to identify the variables eventually correlated to the pressure ulcers (age, anthropometric parameters, nutritional supports, albumin, hemoglobin, calories intake, fluids intake, MM, FM, TBW, ECW, ICW) given that the continuous variables were normally distributed. The logistic regression analysis was used to test the association between pressure ulcer (categorical variable) and the nutritional parameters, adjusting for confounding variables selected among those with p < 0.1 at univariate analysis. Since MAC and MAMC as well as hemoglobin and albumin may be closely linked, to avoid that the one disappearing over the other in the regression due to this, we performed three models including, in the Model I, MAC and hemoglobin, in the Model II, MAMC and hemoglobin and, in the Model III, MAC and albumin. Furthermore, the area under the receiver operating characteristic (ROC) curve was used to analyse the capacity of albumin to predict pressure ulcers and mortality formation. We estimated 5-months cumulative mortality rate stratified by albumin levels using the Kaplan–Meier method. We used Pearson’s χ^2^ test or Mantel extension test for trend for the analysis of discrete variables. Significant differences were assumed to be present at p < 0.05 (two-tailed). All comparisons were performed using SPSS 20.0 for Windows (S. Wacker Drive, Chicago, Illinois 60606, USA).

## Results

### Characteristics of the whole population

Table 1 shows the characteristics of the study population. Among the participants, 8 patients reported a traumatic brain injury, 11 had an ischemic stroke, 8 an hemorrhagic stroke and the remaining had an anoxia event or other. 17 individuals had spastic hypertonia, 13 had constipation, 27 had placed a percutaneous endoscopic gastrostomy (PEG) for enteral nutrition.

**Table 1:** Characteristics of the whole population

Participants (n = 30)	Media ± SD
Variables	
Period of disease (months)	43 ± 39
Age (years)	61 ± 15
Body weight (kg)	52 ± 8
BMI (kg/m^2^)	20 ± 3
Triceps skinfold(cm)	1.28 ± 0.3
Mid arm circumference (cm)	24 ± 2
Mid-arm muscle circumference (cm)	20 ± 2
PA (°)	2.90 ± 0.66
TBW (%)	55 ± 8
ECW (%)	66 ± 6
ICW (%)	33 ± 6
FFM (%)	72 ± 11
FM (%)	27 ± 11
RMR (kcal)	1094 ± 77
Energy expenditure (kcal)	1249 ± 173
Calories intake (kcal)	1724 ± 327
Glucose (mg/dl)	95 ± 26
Creatinine (mg/dl)	0.7 ± 0.2
Calcium (mg/dl)	9.1 ± 0.5
White blood cells (×10^6^/μL)	9.1 ± 4.2
Red blood cells (×10^6^/μL)	4.6 ± 1.6
Hemoglobin (g/dl)	12 ± 2
Iron (mcg/dl)	48 ± 21
Transferrin (mg/dl)	142 ± 96
Albumin (g/dl)	3.3 ± 0.4
Prealbumin (mg/dl)	18 ± 4

*BMI* body mass index, *ECW* extracellular water, *FFM* free fat mass, *FM* fat mass, *ICW* intracellular water, *PA* phase angle, *RMR* resting metabolic rate, *SD* standard deviation, *TBW* total body water

9 patients had pressure ulcers at basal observation (also the total ulcers number was 9) and 11 patients had pressure ulcers at the end of the study (total ulcers number was 13). In particular, only 1 patient reported a Stage II trochanter pressure sores while all other had stage I ulcers at the heel.

### Characteristics of the population according to the albumin levels

When we categorized the population according to albumin levels, only a significant difference of hemoglobin concentration was found (p = 0.01 among groups; Table 2). Among those with low albumin level (group III), 4 patients died and only one patient died among those in the group II. There was no difference in the medications use or other prevalence between groups (data not shown).

**Table 2:** Characteristics of the population according to albumin levels

Variables	Group I (n = 10)	Group II (n = 14)	Group III (n = 6)	p	p (post-hoc analysis)
Albumin levels (g/dl)	3.7 ± 0.2	3.2 ± 0.1	2.6 ± 0.4		
Duration of disease (months)	48 ± 50	45 ± 34	12 ± 3	0.530	ns
Age (years)	56 ± 17	63 ± 12	76 ± 11	0.067	ns
BMI (kg/m^2^)	18 ± 3	21 ± 3	20 ± 4	0.173	ns
Triceps skinfold (cm)	1.2 ± 0.3	1.3 ± 0.3	0.9 ± 0.2	0.084	ns
Mid arm circumference (cm)	23 ± 2	24 ± 2	22 ± 2	0.179	ns
Mid-arm muscle circumference (cm)	19 ± 1	20 ± 2	19 ± 1	0.601	ns
PA (°)	3.1 ± 0.5	2.7 ± 0.5	2.7 ± 1.2	0.306	ns
TBW (%)	54 ± 6	54 ± 8	59 ± 12	0.569	ns
ECW (%)	64 ± 5	68 ± 5	69 ± 11	0.211	ns
ICW (%)	35 ± 5	31 ± 5	30 ± 11	0.204	ns
FFM (%)	70 ± 9	71 ± 11	75 ± 15	0.746	ns
FM (%)	29 ± 9	28 ± 11	24 ± 15	0.746	ns
RMR (kcal)	1092 ± 61	1088 ± 73	1060 ± 103	0.753	ns
Energy expenditure (kcal)	1263 ± 170	1255 ± 170	1088 ± 97	0.176	ns
Calories intake (kcal)	1597 ± 240	1691 ± 297	1883 ± 404	0.279	ns
Glucose (mg/dl)	91 ± 34	96 ± 22	101 ± 19	0.830	ns
Creatinine (mg/dl)	0.68 ± 0.18	0.73 ± 0.18	0.73 ± 0.45	0.895	ns
Calcium (mg/dl)	9.3 ± 0.5	8.9 ± 0.6	8.8 ± 0.2	0.243	ns
Hemoglobin (g/dl)	13.8 ± 1.6	12.3 ± 1.6	9.7 ± 1.2	0.001	I vs III 0.001 II vs III 0.025
Iron (mcg/dl)	60 ± 26	45 ± 16	28 ± 9	0.064	ns
Transferrin (mg/dl)	216 ± 14	191 ± 52	144 ± 16	0.207	ns
Prealbumin (mg/dl)	16 ± 1	20 ± 2	16 ± 12	0.338	ns

### Association between biochemical/anthropometric indexes and pressure ulcers

At univariate (Table 3, showing only the factors significatively correlated to pressure ulcer) and logistic regression (Table 4) analysis, after adjusting for confounding factors, pressure ulcers were inversely associated with both MAC (Model I and III) and MAMC (Model II) (with MAC p = 0.04, beta = −0.89 Model I; with MACM p < 0.050; beta = −1.29 Model II; with MAC p = 0.02; beta = −0.98 Model III) as well as with both haemoglobin (p = 0.04, beta −1,1; Model I and Model II) and albumin (p = 0.04, beta −7.91 model III; Table 4). In the logistic analysis, the correlation between pressure ulcer and TSF and caloric intake disappeared.

**Table 3:** Univariate analysis—Person correlation

Variables	Hemoglobin	Albumin	Triceps skinfold	Mid arm circumference	Mid-arm muscle circumference	Calories intake
Pressure ulcers						
r	−0.506	−0.444	−0.316	−0.542	−0.511	0.346
p	0.005	0.020	0.095	0.002	0.005	0.066

*r* coefficient of correlation, *p* probability

**Table 4:** Logistic regression analysis

Dependent variable Presence of pressure ulcers	B	SE	Exp(B)	p
I Model				
Hemoglobin	−1.177	0.572	0.308	0.040
Calories intake	0.003	0.002	1.003	0.212
MAC	−0.899	0.444	0.407	0.043
II Model				
Hemoglobin	−1.114	0.542	0.328	0.040
Calories intake	0.003	0.002	1.003	0.178
MAMC	−1.298	0.666	0.074	0.050
III Model				
Albumin	−7.91	3.924	0.000	0.044
Calories intake	0.004	0.003	1.000	0.159
MAC	−0.989	0.428	0.372	0.021

Dependent variable: pressure ulcers

*SE* standard error, *P* probability

**Fig. 1:**
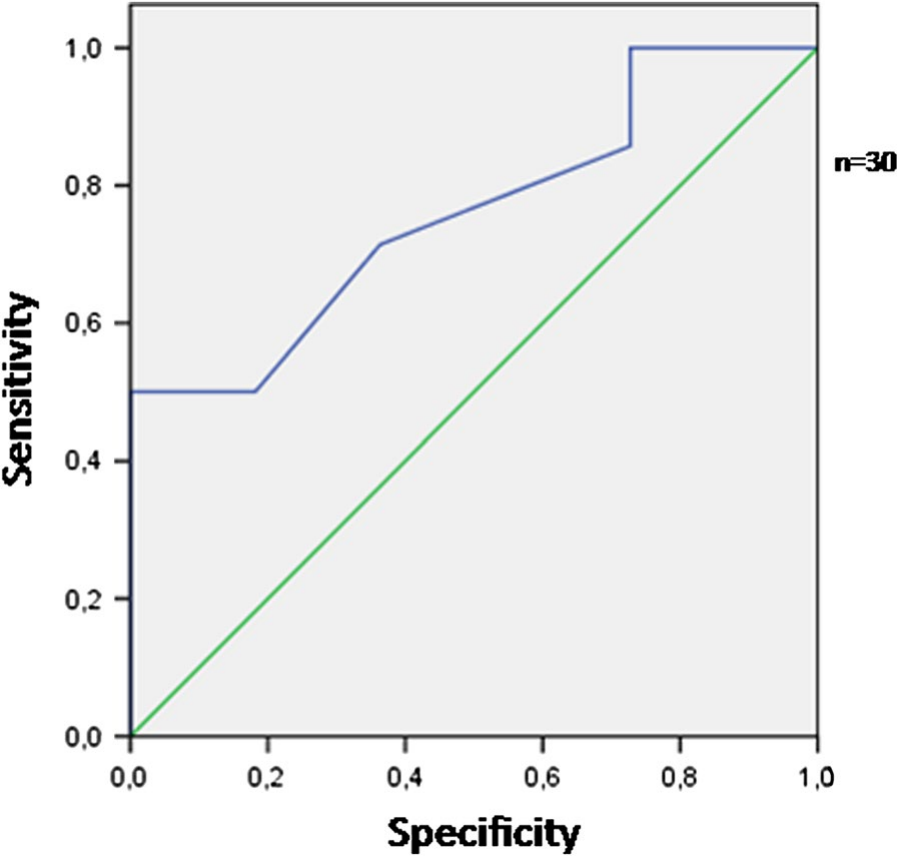
The area under the ROC curve for albumin to predict pressure ulcers

**Fig. 2:**
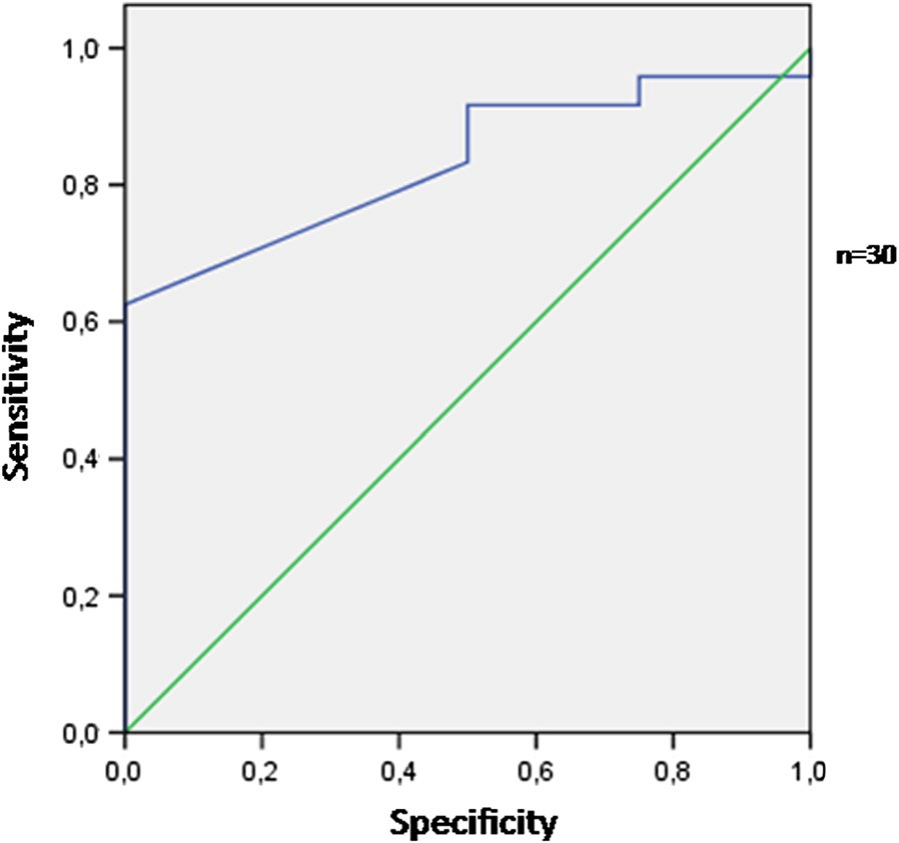
The area under the ROC curve for albumin to predict mortality

The area under the ROC curve for albumin to predict the pressure ulcer formation was 0.76 (SE = 0.09; p = 0.029; lower limit 0.57, higher limit 0.94) (Fig. 1). An albumin concentration equal to 3.1 g/dl achieved adequate sensitivity (72 %) to predict pressure ulcer. The area under the ROC curve for albumin to predict mortality was 0.83 (SE = 0.08; p = 0.036; lower limit 0.67, higher limit 0.99) (Fig. 2). An albumin concentra tion equal to 2.8 g/dl achieved satisfactory sensitivity (75 %) to predict mortality.

### Albumin and short-term mortality prediction

**Fig. 3:**
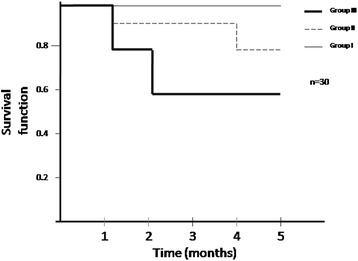
Five-months Kaplan–Meier survival curves stratified by albumin

Short-term mortality during a 5-months period was analyzed. Figure 3 depicts the 5-months cumulative curves of mortality stratified by albumin (log Rank (Mantel-Cox) χ^2^ test = 6.47; df 2; p = 0.039). MCS patients with lower serum albumin had significantly higher short-term mortality than those with higher serum albumin.

## Discussion

In this population we demonstrated an inverse association between some nutritional depletion indicators such as albumin and haemoglobin levels, as well as indexes of skeletal muscle wasting (represented by MAC and MACM) and pressure ulcers. Furthermore, a low albumin concentration (<3.1 g/dl) predicted pressure ulcer formation as well as mortality. Particularly, we found that MCS patients with lower serum albumin had significantly higher short-term mortality than those with higher serum albumin. This is an unprecedented finding, never investigated to date in patients with the long-term consequences of traumatic and non-traumatic acquired brain injury. To our knowledge, limited research has been conducted studying MCS patients, especially the factors associated with pressure ulcers and mortality have never been studied in this particular population.

A low serum albumin level is yet reported to be a predictor of mortality in most populations [8–15] and, as expected, we found that albumin predicts short-term mortality also in MCS patients. Furthermore, it has long been recognised that pressure ulcers are a major cause of morbidity, mortality as well as an healthcare burden globally. Thus, our investigation could have relevant clinical implications.

As suggested by albumin, prealbumin and phase angle, in this investigation the overall population was moderately malnourished (Table 1). However, only albumin, haemoglobin, MAC and MAMC were associated with pressure ulcer (Table 4) and only albumin predicted pressure ulcer formation and mortality (Figs. 1, 2, 3). Pressure ulcer prevalence varies by setting [30–32]. In line with these investigations, we found, at the beginning and end of the study, a prevalence of 30 and 43 % respectively (Results section).

Nevertheless serum albumin has been shown to be associated with pressure ulcers and mortality in different clinical settings [8–15, 33–35], it is well accepted that the serum albumin level could be affected by a variety of factors and could represent an inflammatory marker [36]. Malnourished patients most frequently have inflammation [37, 38]. Nutritional status is one of the main factors involved, but it is not the only one influencing changes in serum albumin [36–38]. In fact in our study, protein and calories intake were adequate (rather exceeding energy needs, Tables 1 and 2). All these facts could suggest the presence of specific mechanisms associated to the reduced biochemical and anthropometric nutritional indicators rather than an inadequate nutritional treatment. Our finding could imply the presence of a syndrome of cachexia, that is characterized by increased cell signalling molecules and inflammation, without the presence of underfeeding [39]. This is confirmed by the fact that there is currently no clear evidence that a nutritional intervention can efficaciously prevent or treat pressure ulcers [40]. In this context, the association between pressure ulcers and both MAC and MACM could confirm the link between pressure ulcer and cachexia [37]. In the light of these concepts, we believe that our study is important since we investigated a well controlled population under the nutritional intake viewpoint.

Furthermore, our study could have an impact on the complexity of the management of patients with severe brain injury, especially in the presence of difficulties of taking accurate weight and height measurements [22]. In fact, a plethora of literature underlined the importance of assessing the arm circumference to identify children with malnutrition at high mortality risk [41, 42]. However, recently it has been showed that MAC may be considered as a simpler alternative to BMI assessment in malnourished individuals [43].

In addition, our study is relevant as it has demonstrated that MCS patients may have a possible perception of suffering [7]. Therefore, independently from various views on end-of-life issues coming out in the context of CDC patients, pressure ulcers may be considered an important ethical problem worthy of attention.

This study has several limitations that must be addressed. The sample size is small. We enrolled patients with a rare clinical condition, as in other investigations [44]. However, the prospective design gives strength to this study and the statistical analysis is adequate. The investigation was carried out on representative samples of both genders who came from a large area of Italy, potentially increasing the generalizability of our findings from a geographical perspective. Of course other known contributing factors for pressure ulcer development like moisture, activity, mobility, friction/shear could have a role in pressure ulcer development and not only malnutrition and several methodologies are known to prevent pressure ulcers. However, this study was not designed to address these factors. Another limitation is that we did not assessed inflammatory mediators as cytokines. Furthermore, we did not perform the Cox proportional hazard models to assess the association between putative confounders and mortality due to the low number of events (only 4 patients died) in this population and in line with other investigations [45]. However, there was no difference in age, duration of disease or BMI among groups (Table 2). Finally, our study was not designed to address the association between pressure ulcers and mortality since we would like only to emphasize the role of some nutritional parameters, and in particular that of albumin, in patients at high mortality and pressure ulcers risk and to suggest the need to explore the mechanisms underlying the cachexia syndrome.

## Conclusion

Limited research has been conducted studying the factors associated with mortality and pressure ulcers in MCS patients. Hypoalbuminemia, regardless of its cause, is a negative prognostic index in MCS patients, which should not go ignored. Furthermore, for the first time, a simple anthropometric parameter as MAC was showed to be associated with pressure ulcers development suggesting the link between pressure ulcer and cachexia rather than underfeeding. Thus, in these patients the nutritional treatments could be optimized but other mechanisms leading to hypoalbuminemia and malnutrition need to be yet explored. A long-term prospective study is required to confirm our results.

## Abbreviations

CDC: chronic disorders of consciousness
MCS: minimal conscious state
MAC: mid-arm circumference
MAMC: mid-arm muscle circumference
TSF: triceps skin fold thickness
MCHC: mean corpuscular hemoglobin concentration
MCV: mean corpuscular volume
